# Supplementary material for: A UMLS-based spell checker for natural language processing in vaccine safety
Source: BMC Med Inform Decis Mak. 2007 Feb 12;7:3. doi: 10.1186/1472-6947-7-3 (PMC1805499; doi:10.1186/1472-6947-7-3)
Supplement: Additional file 13 — RAP application directory. Sets up directory for RAP (RDF application for PHP) source code [file 1472-6947-7-3-S13.gz › rap/doc/phpdoc/resModel/ResResource.html]

xml version="1.0" encoding="iso-8859-1"?


Docs For Class ResResource


## Class ResResource

Description

Description |
Descendents
| Methods (details)

An RDF Resource.

Resource instances, when created, are associated with a specific model. They support a range of methods, such as getProperty() and addProperty() which will access or modify that model. This enables the programmer to write code in a compact and easy style.

History:

- access: public
- author: Daniel Westphal <at d-westphal dot de mail at d-westphal dot de>
- version: V0.9.1

Located in /resModel/ResResource.php (line 25)

```
Object
   |
   --Node
      |
      --Resource
         |
         --ResResource
```

Direct descendents

Description |
Descendents
| Methods (details)

| Class | Description |
| --- | --- |
| OntResource | Provides a common super-type for all of the abstractions in this ontology representation package. |
| ResContainer | An RDF Container. |
| ResList | Implementation of an rdf:Collection (rdf:List) Provides a convenience encapsulation for lists formed from chains of RDF statements arranged to form a head/tail cons-cell structure. |
| ResProperty | An RDF Property. |

Method Summary

Description |
Descendents |
Methods (details)

ResResource
ResResource
(string $uri)

object ResResource
addProperty
(ResResource $property, ResResource/ResLiteral $object)

boolean
equals
(object resource $that)

object Model
getAssociatedModel
()

boolean
getIsAnon
()

object ResResource
getProperty
(ResResource $property)

object ResResource
hasProperty
(ResResource $property, [ResResource $value = null])

ResIterator
listProperties
([ResResource $property = null])

object ResResource
removeAll
([object resource $property = null])

object ResResource
removeProperties
()

void
setAssociatedModel
(object Model &$model)

void
setIsAnon
(boolean $isAnon)

void
setURI
(string $uri)

Methods

Description |
Descendents |
Methods (details)

Constructor ResResource (line 49)

Constructor You can supply a uri

- access: public

ResResource

ResResource
(string $uri)

- string
  $uri

addProperty (line 100)

Add a property to this resource.

A statement with this resource as the subject, p as the predicate and o as the object is added to the model associated with this resource.

- access: public

object ResResource

addProperty
(ResResource $property, ResResource/ResLiteral $object)

- ResResource
  $property
- ResResource/ResLiteral
  $object

---

Redefined in descendants as:

- OntResource::addProperty()
  : Add a property to this resource.

equals (line 178)

Checks if the resource equals another resource.

Two resources are equal, if they have the same URI

- access: public

boolean

equals
(object resource $that)

- object resource
  $that

---

Redefinition of:

Resource::equals()
:   Checks if the resource equals another resource.

getAssociatedModel (line 74)

Get the reference to the assocoated model.

- access: public

object Model

getAssociatedModel
()

getIsAnon (line 154)

Determine whether this resource is an anonymous resource

- access: public

boolean

getIsAnon
()

getProperty (line 128)

Answer some statement (this, p, O) in the associated model.

If there are several such statements, any one of them may be returned. If no such statements exist, null is returned.

- access: public

object ResResource

getProperty
(ResResource $property)

- ResResource
  $property

hasProperty (line 141)

Determine whether this resource has any values for a given property.

- access: public

object ResResource

hasProperty
(ResResource $property, [ResResource $value = null])

- ResResource
  $property
- ResResource
  $value

listProperties (line 114)

List all the values with the property p as statements in an array.

- access: public

ResIterator

listProperties
([ResResource $property = null])

- ResResource
  $property

removeAll (line 194)

Delete all the statements with predicate p for this resource from its associated model.

- access: public

object ResResource

removeAll
([object resource $property = null])

- object resource
  $property

removeProperties (line 209)

Delete all the properties for this resource from the associated model.

- access: public

object ResResource

removeProperties
()

setAssociatedModel (line 61)

Sets the reference to the assocoated model.

- access: public

void

setAssociatedModel
(object Model &$model)

- object Model
  $model

setIsAnon (line 165)

Set whether this resource is an anonymous resource

- access: public

void

setIsAnon
(boolean $isAnon)

- boolean
  $isAnon

setURI (line 85)

Sets the URI of this resource

- access: public

void

setURI
(string $uri)

- string
  $uri

#### Inherited Methods


Inherited From Resource

> Resource::Resource()  
> Resource::equals()  
> Resource::getLabel()  
> Resource::getLocalName()  
> Resource::getNamespace()  
> Resource::getURI()  
> Resource::toString()

Inherited From Object

> Object::toString()

Documentation generated on Thu, 7 Jul 2005 13:43:48 +0200 by phpDocumentor 1.3.0RC3
